# The burden of coronary heart disease in simultaneous pancreas-kidney transplantation: coronary angiography as a diagnostic method for all? - a retrospective study

**DOI:** 10.1590/2175-8239-JBN-2021-0156

**Published:** 2022-02-28

**Authors:** Joana Marques, Luísa Pereira, Ana Messias, Nuno Fonseca, Patrícia Cotovio, Aníbal Ferreira, Fernando Nolasco

**Affiliations:** 1 Centro Hospitalar de Lisboa Central, Serviço de Nefrologia, Lisboa, Portugal.; 2 Centro Hospitalar Universitário do Algarve, Serviço de Nefrologia, Faro, Portugal.; 3 Hospital Garcia de Orta, Serviço de Nefrologia, Almada, Portugal.; 4 Nova Medical School, Hospital Curry Cabral, Serviço de Nefrologia, Lisboa, Portugal.

**Keywords:** Diabetes Mellitus, Type 1, Kidney Failure, Chronic, Simultaneous Pancreas-Kidney Transplantation, Coronary Artery Disease, Coronary Angiography, Diabetes Mellitus Tipo 1, Falência Renal Crônica, Transplante de Pâncreas-Rim Simultâneo, Doença da Artéria Coronariana, Angiografia Coronária

## Abstract

Introduction: Type 1 diabetes mellitus is associated with an increased risk of coronary artery disease, which is frequently asymptomatic. This risk increases significantly in those with nephropathy. In selected patients, simultaneous pancreas-kidney transplantation is the renal and pancreatic replacement therapy of choice, as it increases longevity and stabilizes diabetic complications. Despite essential, universal screening protocols are still controversial for coronary artery disease in this population. Methods: We retrospectively analysed 99 simultaneous pancreas-kidney recipients from our centre from 2011 to 2018 and selected 77 patients who underwent coronary angiography during the pre-transplant evaluation. Our aim was to identify potential risk factors associated with significant lesions on coronary angiography. Results: Almost half of our cohort of 77 candidates submitted to coronary angiography had coronary artery disease. Of these, nearly 30% underwent revascularization, although only one of them reported symptoms of myocardial ischemia. In a univariate analysis, the presence of smoking habits was the only risk factor for coronary artery disease. We also found that 20 or more years of type 1 diabetes mellitus was significantly associated with the presence of coronaropathy. Discussion: Selection of diabetic candidates with acceptable cardiac risk before simultaneous pancreas-kidney transplantation is imperative. Given the impact of a correct diagnosis and a low procedural risk, we defend the routine use of coronary angiography as the initial screening method for coronary artery disease in this population. Particularly care must be taken in evaluating asymptomatic patients with long-term type 1 diabetes mellitus and smokers.

## Resumo

Introdução: O diabetes mellitus tipo 1 está associado ao risco aumentado de doença arterial coronariana, que é frequentemente assintomática. Este risco aumenta significativamente em pessoas com nefropatia. Em pacientes selecionados, o transplante de pâncreas- rim simultâneo é a terapia substitutiva, renal e pancreática, de escolha, pois aumenta a longevidade e estabiliza complicações diabéticas. Apesar de essenciais, protocolos de triagem universais ainda são controversos para doença arterial coronariana nesta população. Métodos: Analisamos retrospectivamente 99 receptores de pâncreas-rim simultâneo de nosso centro, de 2011 a 2018, e selecionamos 77 pacientes que realizaram angiografia coronária durante avaliação pré-transplante. Nosso objetivo foi identificar fatores de risco potenciais associados a lesões significativas na angiografia coronária. Resultados: Quase metade de nossa coorte de 77 candidatos submetidos à angiografia coronária apresentou doença arterial coronariana. Destes, quase 30% foram submetidos à revascularização, embora apenas um tenha relatado sintomas de isquemia miocárdica. Em uma análise univariada, a presença do hábito de fumar foi o único fator de risco para doença arterial coronariana. Também descobrimos que 20 ou mais anos de diabetes mellitus tipo 1 estavam significativamente associados à presença de coronariopatia. Discussão: A seleção de candidatos diabéticos com risco cardíaco aceitável antes do transplante de pâncreas-rim simultâneo é imperativa. Dado o impacto de um diagnóstico correto e baixo risco de procedimento, defendemos o uso rotineiro da angiografia coronária como método de triagem inicial para doença arterial coronariana nesta população. Deve-se ter um cuidado especial na avaliação de pacientes assintomáticos com diabetes mellitus tipo 1 de longa duração e fumantes.

## Introduction

Diabetes is a leading risk factor for coronary artery disease (CAD). Once diagnosed with CAD, diabetic patients have a considerably worse prognosis than non-diabetics^1^.They usually present diffuse and severe CAD with massive atherosclerotic plaques consisting of bulky lipid cores, which induce high rates of remodelling in the affected vascular segment and thin but highly inflammatory fibrous caps that make them more vulnerable to rupture^2^. Moreover, as the process develops faster and earlier and autonomic neuropathy often coexist, the patient may remain asymptomatic for years^2^. Consequently, mortality from cardiovascular (CV) disease remains 2 to 8 times higher in diabetic patients compared with the general population^3^. Those with type 1 diabetes mellitus (T1DM) are at a particularly high risk of premature CAD, with up to 35% dying of CAD by the age of 55 years. The risk of death increases if they have nephropathy^4^.

Simultaneous pancreas-kidney transplantation (SPKT) is the preferred option for renal and pancreatic replacement therapy in selected patients with end-stage renal disease (ESRD) and T1DM. A successful SPKT restores optimal glycemic control, thereby stabilizing other T1DM secondary complications such as retinopathy, neuropathy, CV disease and life-threatening hypoglycaemic episodes. It consequently improves patient longevity - mainly by decreased progression of CAD - and quality of life^3,4,5^.Nonetheless, despite great advances, SPKT is still a complex procedure, which associated with significant morbidity rates. SPKT recipients have a high risk of cardiac ischemia following surgery and present a risk of perioperative CV events that may exceed 10%.^6^ In addition, previous CV risk is substantially worsened by chronic use of immunosuppressants^7^. Ultimately, CV is a leading cause of death after SPKT^6^.

On his recent study, St. Michel et al.^6^ showed that more than two-thirds of SPKT candidates who underwent coronary angiography (CAG) before transplantation had CAD. This suggests that critical evaluation and selection of SPKT candidates potentially minimize serious adverse events and improve outcomes^3^. Preoperative CV assessment is also mandatory to select patients who may maximally benefit from SPKT^8,9^. Nevertheless, an universal screening protocol for CAD in SPKT candidates is still lacking, and it is surprisingly uncertain whether a non-invasive or an invasive strategy should be performed in this population.

We present a single center retrospective study including SPKT candidates with a routine pre-transplantation CAG. Our aim was to identify predictive risk factors associated with the presence of CAD on CAG, and thus contribute to the elaboration of a screening algorithm for this population.

## Subjects and Methods

### Patients

We retrospectively analyzed 99 SPKT recipients at our center from January 2011 to December 2018. A cohort of 78 patients who underwent CAG as a screening method for CAD during the pre-transplant study period was enrolled in this study. One patient was excluded due to lack of data.

We evaluated candidates’ demographic and clinical information including age, gender, duration of T1DM, and, if applicable, dialysis vintage, presence of secondary complications of T1DM, and concurrence of other CV risk factors such as obesity, hypertension, dyslipidemia, and tobacco abuse. We defined obesity as a body mass index >30 kg/m^2^ and hypertension or dyslipidemia as the treatment with antihypertensive drugs or statins, respectively. Data on serum glycated hemoglobin and lipid levels were obtained from patients’ medical records immediately before CAG procedure.

### Coronary artery disease

The existence of CAD was considered when one or more coronary lesions were revealed by CAG. Whenever a lesion was treated, a significant CAD was assumed.

Of the 77 patients, those with non-invasive positive tests, such as a pharmacological stress echocardiogram (PSE) or a myocardial perfusion scintigraphy (MPS), were considered to have coronariopathy.

### Statistical analysis

Continuous and categorical variables are presented as mean ± standard deviation (SD) and frequency (percentage [%]), respectively. Categorical variables were compared using the Chi-square or the Fisher test. For comparison between groups of patients with and without CAD on CA we used analysis of variance (ANOVA). Statistical significance was set at *p* value <0.05.

Statistical analyses were performed using the SPSS statistics version 23.0 for Windows (IBM Corp., Armonk, NY, USA).

### Ethics

Informed consents were obtained to initiate the evaluation of the candidate and perform the CAG and the SPKT.

## Results

From January 2011 to December 2018, a total of 99 SPKT were performed in our institution. Of these, 77.8% (N=77) presented complete data, were submitted to the preoperative CAG as a screening test for CAD, and included in our study.

The mean recipient age was 36 ± 5.9 years (range: 21-53). Eight patients (10.4%) were aged 50 or older. Forty-nine patients (63.6%) were male. The majority was Euro-Caucasian (N= 67; 87.0%).

All SPKT candidates had T1DM, with a mean duration of disease of 25 ± 5.4 years (range: 13-49). Sixty-three patients (81.8%) had T1DM for 20 years or more. Medium serum glycated hemoglobin before CAG was 9.5 ± 1.8% (range: 5.1-12.9). All candidates had nephropathy, the majority with ESRD (N=75; 97.4%), with a dialysis vintage of 37 ± 44.7 months (1-192). The remaining 2 SPKT (2.6%) were pre-emptive. Also, all patients had at least another T1DM secondary complication: 97.4% (N=75) had retinopathy, 42.8% (N = 33) had peripheral neuropathy, 31.2% (N = 24) had dysautonomia, 19.5% (N = 15) had peripheral arterial disease, and 6.5% (N=5) had cerebrovascular disease. Only one patient (1.3%) reported episodes suggestive of angina pectoris (Table 1).

**Table 1:** Characterization of SPKT candidates according to T1DM complications, coexistence of other cardiovascular factors, and correlation with the presence of lesions on CAG

Type of lesion on CAG	Mean age	Male	Microvascular disease	Macrovascular disease	Smoke	Positive PSE (N=14)	Positive MPS (N=17)	Total
Significant	35 ± 7,6	5	11	2	8	3	0	11
Not significant	36.5 ± 7.4	20	26	6	16	2	6	26
Total	36 ± 7.3	25	37	8	24	5	6	37

Y: years. NA: not applicable; NS: non significant. ESRD: end stage renal disease.

With regard to the remaining CV risk factors, most patients had arterial hypertension (87%, N = 67), 32.8% (N= 22) of whom were medicated with 3 or more antihypertensive drugs, and 55.8% (N=43) had dyslipidemia. Thirty seven patients (48.1%) had active smoking habits and 4 patients (5.2%) were obese (Table 1).

Of those on dialysis, CAG was preformed 11 ± 486 months after its initiation. CAG identified at least one injury in 48.1% (N = 37) of SPKT candidates. Eleven candidates (14.3%, but 29.7% of those with an injury) underwent intervention for hemodynamically significant CA. None of treated patients had angina pectoris.

Thirty-one candidates (40.3%) had done a PSE (N = 14; 18.2%) or a MPS (N= 17; 22.1%) before CAG. Two candidates (2.6%) did both. Half of the patients with a pre-CAG PSE (N=7) had a positive result for myocardial ischemia. CAD was not confirmed by CAG in 2 of them (28.5%). On the other hand, 2 patients with a negative PSE showed not significant CAD on CAG (28.5% out of 7 with a negative test). Six of the patients with a pre-CAG MPS (35.3%) presented a positive test for ischemia, with no confirmation of CAD on CAG in half of them (N = 3). Five candidates with a clean MPS (45.5% of the negative group) had CAD diagnosed by CAG; 2 of them were revealed to be significant. The 2 patients with non-invasive tests showed a negative PSE but a MPS positive for ischemia. One of them presented no significant CAD on CAG.

Univariate analysis showed that the only distinguishing feature between the group of patients with and without CAD revealed by CAG was smoking (*p*=0.005). None of the other CV risk factors nor the presence of specific T1DM secondary complications were related to the presence of CAG lesions in our population of SPKT candidates. We also concluded that T1DM with 20 or more years of evolution was significantly associated with coronariopathy (identifiable by any diagnostic test) (*p*=0.048) (Table 1).

## Discussion

Starting in 2007, our institution observed a large increase in SPKT volumes. Over the past decade, the surgical technique has consistently improved, but the complexity of SPKT candidates is a growing challenge. Nowadays, candidates are older, more of them are obese, and frequently have a higher burden of comorbidities^6,10^. Considering the CV impact on SPKT recipients, it seems imperative to detect and treat significant CAD before transplantation to limit the selection to those with acceptable cardiac risk^8,9^. As we recognize the accelerated progression of atherosclerosis in T1DM patients, we agree that none of our candidates can truly be classified as “low risk” for CAD based on traditional CV risk factors^11^. Because silent ischemic heart disease is typical of T1DM, we do not rely on symptoms as the sole criterion for preoperative CAG.

The assessment of cardiac disease in SPKT candidates remains surprisingly understudied. When choosing between non-invasive stress testing and invasive CAG, there are few and conflicting data supporting the use of methods other than CAG^11^. While some authors believe screening diabetic transplant candidates with CAG is justifiable, others argue that this invasive method should only be used in patients with a very high pre-test probability, such as those with previous cardiac events, ischemic symptoms, long standing diabetes, or significant ischemic burden on stress imaging^8,11,12^. However, the inaccuracy of noninvasive imaging must be weighed against the low but non-negligible risk of procedural complications with CAG^12^.

By recognizing the risk of CAD of T1DM patients and taking into account all the above-mentioned facts, we use a rigorous approach to cardiac evaluation in the pre-transplant setting at our center. All SPKT candidates perform an electrocardiogram, a chest radiograph, and an echocardiogram. In addition, if there are no contraindication or ESRD has not yet been reached, patients are also proposed for invasive CAG to evaluate the presence and extent of CAD. If CAD proves significant, patients are treated before SPKT. As no major complications have occurred after CAG since this approach was implemented, CAG seems to be a safe procedure. After being on active list we do not routinely repeat the CAG, as the procedure to SKPT is usually expeditious.

This study showed that approximately half of the SPKT candidates had asymptomatic CAD and consequently nearly 30% of these underwent revascularization. For every 7 SPKT candidates submitted to pre-transplant CAG, one needed coronary intervention. Because one missed diagnosis can have a great impact, we believe that this data justify the routine utilization of CAG as an initial screening method for CAD in SPKT candidates.

Fewer than half of our patients had performed a non-invasive stress test prior to invasive CAG. The use of a PSE or MPS was arbitrarily decided. Accordingly, we recognize that no significant interpretations can be derived from this. However, we highlight the low concordance of coronariopathy detected by non-invasive and invasive ischemic tests in our population. Two out of 7 and 5 out of 11 SPKT candidates with negative DSE and MPS, respectively, had evidence of CAD on routine pre-transplant CAG. In addition, 2 of the patients with negative MPS were treated for significant CAD after it was detected by CAG. Table 2 lists the SPKT with CAD detected by CAG.

**Table 2:** Characterization of SPKT candidates with coronary artery disease revealed by CAG

	Total (N= 77)	Lesions on CAG (N=37)	No lesions on CAG (N=40)	p-value
T1DM vintage (years) T1DM > 20 years	25 ± 5.4 (13-43) 81.8% (63)	29± 6.1 (13-43) 97.3% (36)	22 ± 4 (17-36) 67.5% (27)	NS 0.048
Medium serum glycated hemoglobin	9.5% ± 1.8 (5.1-12.9)	8.9% ± 1.4 (6-12.1)	9.2 ±1.9 (5-12.9)	NS
ESRD Dialysis vintage (months)	97.4% (75) 37 ± 44.7 (1-192)	97,3% (36) 31.6± 21.4 (11-92)	97,5% (39) 44.5 ± 40.9 (1-192)	NS
Retinopathy	97.4% (75)	97.3% (36)	97.5% (39)	NS
Neuropathy	42.8% (33)	32.4% (12)	52.5% (21)	NS
Dysautonomia	31.2% (24)	21.6% (8)	40% (16)	NS
Peripheral arterial disease	19.6% (15)	13.3% (5)	25% (10)	NS
Cerebrovascular disease	6.5% (5)	8.1% (3)	5% (2)	NS
Angor	1.3% (1)	0	2.5% (1)	NS
Arterial hypertension 3 or more agents	87% (67) 28.6% (22)	89.2% (33) 32.4% (12)	85% (34) 25% (10)	NS
Dyslipidemia	55.4% (43)	48.6% (18)	62.5% (25)	NS
Smoking habits	48% (37)	64.9% (24)	32.5% (13)	0,005
Obesity	5.2% (4)	2.7% (1)	7.5% (3)	NS

DM: Diabetes Mellitus; HD: hemodiálise; H: Homem. M: Mulher. S: sim. N: não. NF: não feito. DP: diálise peritoneal. EEF: ecocardiograma com estresse farmacológico. CPM: cintilografia de perfusão miocárdica.

The single risk factor for CAD in our study was smoking, contradicting previous studies that indicated that age was the most significant factor.^10,11^ Duration of T1DM also seemed to be relevant. In our cohort, 20 or more years of T1DM were found to be significantly associated with the presence of coronariopathy and/or CAD. None of the other CV risk factors or the presence of specific T1DM secondary complications were associated with CAD, limiting our desire for a screening algorithm. Nevertheless, the message must be maintained that a clinical history of CV symptomatic disease is not enough to predict the existence of CAD in our population, although it is very important. Particularly, careful evaluation is warranted in asymptomatic patients with long-term T1DM and in smokers.

Our study had some limitations. First, data were retrieved retrospectively and the sample size was small, which undoubtedly limit the power of this cohort study. In addition, as there was no internal comparison group, historic data were gathered for comparison. In doing so, we cannot draw any conclusions between candidates who performed both non-invasive and invasive cardiac evaluations. Further studies, perhaps larger and multicenter, should be encouraged as they could help to offset these limitations and contribute to the development of a screening algorithm for SPKT candidates in the future. Finally, we agree that medium- and long-term follow-up studies are needed to evaluate the effects of pre-transplant candidate selection on post-transplant CV events and survival rates.
